# Epidemiological, Pathological, and Molecular Studies on Sheeppox Disease Outbreaks in Karnataka, India

**DOI:** 10.3390/microorganisms12071373

**Published:** 2024-07-04

**Authors:** Gundallahalli Bayyappa Manjunatha Reddy, Varun Kumar Krishnappa, Chandan Dypasandra Siddalingaiah, Suguna Rao, Shivasharanappa Nayakvadi, Chethan Kumar Harlipura Basavarajappa, Baldev Raj Gualti

**Affiliations:** 1 ICAR-National Institute of Veterinary Epidemiology and Disease Informatics (NIVEDI), Bengaluru 560064, Karnataka, India; 2 Veterinary College, Karnataka Veterinary, Animal and Fisheries Sciences University (KVAFSU), Hebbal, Bengaluru 560024, Karnataka, India; sachinkumark1410@gmail.com (V.K.K.);

**Keywords:** epidemiology, pathology, sheeppox, disease outbreak, *P32* gene, phylogenetic analysis

## Abstract

An epidemiological study spanning twelve years has revealed that sheeppox disease is both widespread and endemic, predominantly surging during the winter and summer seasons. This investigation focused on sheeppox across 11 field outbreaks, involving 889 animals from non-migratory flocks across six districts in Karnataka, in the southern peninsula of India. Among these, 105 animals exhibited clinical signs suggestive of sheeppox, such as lesions on the body, and 95 cases were confirmed through PCR testing. The overall positivity rate for sheeppox stood at 10.68% (95 out of 889 animals). The incidence of sheeppox was notably higher in animals aged between 1 and 2 years and was more prevalent in females. Affected animals displayed symptoms including respiratory distress, weakness, fever, loss of appetite, depression, and various skin lesions ranging from papular to pock lesions across their bodies. There was a significant increase in total leukocyte count, while hemoglobin levels, red blood cell counts, and hematocrit values significantly decreased. On gross examination, sheeppox lesions, varying from vesicular to nodular forms, were predominantly found on hairless areas of the body. Microscopic examination of skin lesions revealed extensive changes, such as hyperkeratosis, parakeratosis, acanthosis, hydropic degeneration, and necrosis of epithelial cells, along with characteristic intracytoplasmic viral inclusions. The lungs exhibited type-II pneumocyte hyperplasia and proliferative bronchiolitis, also with intracytoplasmic inclusions. Confirmation of the sheeppox virus was achieved through PCR and subsequent sequence analysis. Phylogenetic analysis of the full-length *P32* and *RPO30* gene demonstrated homology with sheeppox isolates from various parts of India and neighboring countries, indicating that Indian sheeppox viruses are highly lineage-specific and correlate with the host of origin. Based on these findings, it is recommended to implement a homologous vaccination strategy, utilizing selective host/viral strains to enhance protection in susceptible animals.

## 1. Introduction

Sheep farming plays a crucial role in the livelihoods of marginal and landless farmers, contributing significantly to income generation and improving household nutrition. According to the 2019 livestock census, India has the fourth largest sheep population in the world, numbering 74.26 million, trailing behind China, Australia, Sudan, and Iran. This represents a 10.1% increase from the previous census. Sheep farming contributes approximately 8.36% to the total meat production and yields 40.42 million kilograms of wool. Sheep diseases, particularly those that are highly contagious, pose a significant threat, leading to both immediate and prolonged economic losses for farmers. These losses impact not only the production but also the trade of small ruminants and their by-products [1].

Sheeppox is caused by the sheeppox virus (SPPV) within the *Capripoxvirus* genus of the *Chordopoxvirinae* subfamily and *Poxviridae* family [2]. The World Organization for Animal Health (OIE) has classified sheeppox as a notifiable disease. It is endemic in several regions, including India, Central Asia, parts of China, the Middle East, and Central and Northern Africa [3]. Sheep of all ages, breeds, and sexes are susceptible to sheep pox [4]. The disease manifests with morbidity rates as high as 90% and mortality rates between 5–10%, which can soar to 100% among imported breeds [4]. Clinically, sheeppox presents in two forms: a more severe (malignant) form, particularly in lambs, characterized by high fever, respiratory distress, ocular and nasal discharge, and potentially lethal pox lesions on un-wooled skin, and a less severe (benign) form, typically seen in adult sheep, marked by localized skin lesions, especially under the tail [5].

Given its highly contagious nature, sheeppox necessitates rapid and precise laboratory diagnosis. Polymerase Chain Reaction (PCR) has been developed to detect specific target genes in the Capripoxvirus, including the *P32* gene [6,7], *RPO30* gene, and *GPCR* gene [8,9,10,11]. This study undertakes a comprehensive epidemiological investigation of sheeppox outbreaks, combining molecular epidemiology with the examination of pathological lesions in spontaneous cases.

## 2. Materials and Methods

### 2.1. Passive Disease Outbreak Data Collection

We collected quantitative data on sheep and goatpox disease outbreaks from the Department of Animal Husbandry and Veterinary Services in Karnataka over a 12-year span from 2010 to 2022. These data included the number of outbreaks, cases, and fatalities at the district level, allowing for the analysis of the spatial and seasonal variations of sheeppox and goatpox. The case fatality ratio (CFR) was calculated using the formula: CFR = (Total number of deaths/Total number of affected cases) × 100.

### 2.2. Geographical Study Area and Sample Size

The investigation of outbreaks was conducted in 2020 across selected districts in the state of Karnataka, India. The district’s selection was based on the frequency of reported outbreaks over the last decade, categorizing districts into three levels for sample collection: low (Bengaluru Rural and Bengaluru Urban), medium (Chikkaballapura, Kolar), and high (Tumkur, Chitradurga, Ballary). Field veterinarians assisted in investigating the sheeppox outbreaks in these districts (Table 1).

The sample size for seroprevalence was determined for the finite or large population as per Cochran (1963) formula N = Z^2^ [p (1 − p/e^2^] using the epitool, where N = sample size, Z = 95% confidence level, *p* = 50% proportion (animal unit-level prevalence of 50% was considered as there are no seroprevalence studies on sheeppox and goatpox in India), and e is the precision level (5%). Based on these inputs, a total sample size of 385 was determined (http://epitools.ausvet.com.au/content.php?page=1Proportion, accessed on 30 March 2023) for the target populations in the study region. However, after considering the design effect of 2.0, the total arrived sample size was 770. In the first stage, three districts (High, Medium, and Low density) from Karnataka state were selected based on the small ruminants’ population density, and samples were distributed based on the proportion of sheep and goats in the selected district. In the second stage, the Epiunit/villages with at least 500 small ruminants were identified, and sera samples were collected as per the allotted sample size proportion in 10 randomly selected villages in each district for seroprevalence study.

### 2.3. Clinical Examination and Collection of Samples

Each outbreak was documented for history, flock size, the number of affected and deceased animals, age, and sex. The clinical examinations of affected flocks were conducted with the help of local veterinarians to assess general health and the distribution of pox lesions, which were graded by their number per 5 cm^2^ around the mouth and base of the tail. Lesions were classified as mild (1–2 lesions), moderate (3–4 lesions), or severe (5 or more lesions) per 5 cm^2^. Out of 889 susceptible animals, 105 were suspected of having sheeppox, from which scab and skin biopsy samples were collected in 10% neutral buffered formalin for pathological studies and in virus transport media for PCR analysis. Blood and serum samples were collected from both affected and healthy animals for total leukocyte count (TLC), total erythrocyte count (TEC), hemoglobin (Hb), packed cell volume (PCV), and platelet count, along with various biochemical analyses of the serum samples. The serum samples were submitted for iELISA for the detection of Capripoxvirus antibodies. 

### 2.4. DNA Extraction, Amplification and Sequencing of P32 and RPO30 Gene

DNA extraction from scab and biopsy samples was performed using the QIAamp DNA Mini Kit (Qiagen Pvt. Ltd., New Delhi, India, catalog no. 51306). PCR amplification and sequencing of the *P32* gene employed oligonucleotide primers SGPP 32-F: ACACA GGGGGA TATGA TTTTACC and SGPP 32-R: ATACCG TTTTTCAT TTCGTTAGC, for a partial 237 bp segment of the *P32* gene for preliminary genus-specific confirmation. This was followed by full-length *P32* and *RPO30* gene amplification for phylogenetic analysis [6,11]. The DNA from the vaccine strain sheeppox virus (RF-Strain) maintained at ICAR-NIVEDI, Bengaluru, served as a positive control. The PCR products were purified using the Gene JET Gel Extraction Kit (Thermo Fisher Scientific, Waltham, MA, USA) and sequenced bidirectionally using the Sanger sequencing method (Eurofins, Bengaluru, India). The complete coding region sequences of the *P32* and *RPO30* genes were analyzed using the BLAST-n bioinformatics tool, with the nucleotide sequences submitted to GenBank with accession numbers OQ737021 to OQ737026. 

### 2.5. Phylogenetic Analysis and Multiple Sequence Alignment

For the phylogenetic analysis of the *P32* and *RPO30* gene sequence, we retrieved published Capripoxvirus (CaPV) *P32* and *RPO30* gene sequences from the GenBank database. Among these, five sequences were identified as belonging to both field and vaccine strains of sheeppox isolates from India. The construction of the maximum likelihood phylogenetic tree was based on MUSCLE alignment, utilizing the MEGA-X software, version 10.0.5, with a bootstrap value of 1000 for robust sequence comparison [12]. Additionally, we performed multiple sequence alignments of both nucleotide (nt) and amino acid (aa) sequences for all the sheeppox virus (SPPV) and goatpox virus (GTPV) sequences, using the DNASTAR Lasergene software, version 6.0, to determine the sequence identities and divergences among the strains.

## 3. Results

### 3.1. Retrospective Analysis of Sheeppox Outbreak and Seroprevalences

**Figure 1:**
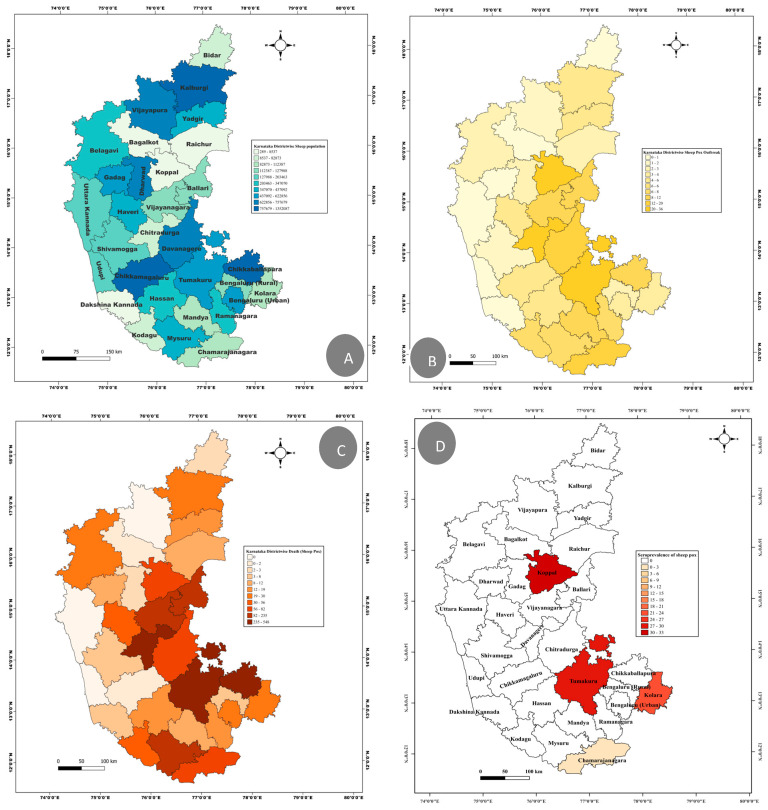
Spatial maps: The sheep population density in Karnataka state province (**A**), sheeppox disease outbreaks density (**B**), number of deaths due to sheeppox (**C**), and seroprevalence of sheeppox disease in different districts of Karnataka (**D**).

Throughout the period of from 2010 to 2022, sheeppox outbreaks were reported in 28 out of 31 districts in Karnataka, highlighting the widespread nature of the disease across the state. The district of Koppala reported the highest number of outbreaks, followed by Tumkur, Davangere, and Chitradurga, indicating areas of particular concern (Supplementary Figure S1). Conversely, the fewest outbreaks were recorded in Dakshina Kannada, Bagalkot, Dharwad, and Bidar, with each district reporting a single outbreak. Notably, no outbreaks were reported in Udupi and Uttar Kannada districts. Tumkur, Davangere, Chickballapur, Bellary, and Mysore were among the districts with a higher number of reported deaths among the affected cases. In contrast, the Vijayapura district, despite experiencing two outbreaks affecting 105 animals, did not report any deaths (Figure 1).

A time trend analysis of the data collected over these twelve years revealed that sheeppox was reported year-round, with a peak in outbreaks occurring between December and May. The most fatalities were observed in February and March. The year 2010 saw the highest number of outbreaks (n = 72), with subsequent peaks in 2016 (n = 34) and 2014 (n = 31). Conversely, 2012 and 2018 each reported the fewest outbreaks (n = 1). The year 2016 had the highest number of reported cases, followed by 2014 and 2010. Interestingly, despite a lower number of outbreaks in 2017 (n = 18), this year witnessed a significant number of deaths (n = 362) compared to previous years (Supplementary Figure S2). The average case fatality rate over this period was 30.92%, with a range from 0 to 100% among the affected animals. This variability underscores the disease’s impact and the importance of continued monitoring and intervention strategies.

The serological data from 772 sera samples revealed the lowest in Chamarajanagar (3%). Whereas, irrespective of population density, the other three districts (Tumakuru, Koppala, and Kolar) revealed high seropositivity for sheeppox disease, ranging from 29 to 31% (Figure 1D).

### 3.2. Epidemiological, Clinical, and Pathological Findings in Sheeppox Outbreaks

In this study, we attended 11 field outbreaks of sheeppox, where 105 cases were initially suspected based on clinical observations, and 95 of these cases were later confirmed to be sheeppox virus (SPPV) infections through PCR testing. The observed average rates for morbidity, mortality, and case fatality were 10.69%, 4.72%, and 45.26%, respectively. A notable finding was the higher morbidity rate in the 1–2-year age group (42.10%), followed by the group below 1 year (31.57%), with the lowest morbidity observed in sheep older than 2 years (26.31%). Additionally, females were more commonly affected than males, with percentages of 57.89% and 42.1%, respectively.

**Figure 2:**
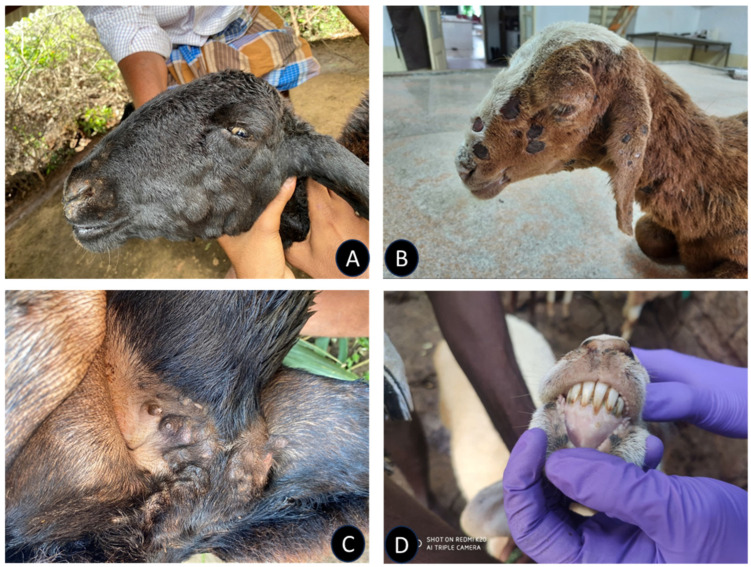
Photograph of sheeppox-affected animal showing pox lesions on head (**A**), face (**B**), mammary gland (**C**), and lips (**D**), ranging from moderate to severe grade lesions.

Clinically, the affected sheep exhibited symptoms such as coughing, respiratory distress, fever ranging between 105–106 °F, and thick mucopurulent nasal discharge, accompanied by pale conjunctival mucous membranes. Pock lesions were prominently found on various parts of the body including the lips, cheeks, nostrils, eyelids, ears, head, neck, inner thigh, tail base, perineum, vulval lips, udder, teats, inguinal region, and scrotum. Additionally, ulcerative lesions were observed on the lingual surface of the lips, gums, tongue, and hard palate (Figure 2).

The severity of pox lesions was markedly greater on hairless regions, particularly the muzzle and the inner aspect of the tail. The skin nodules presented as greyish-white masses, varying from round to irregular shapes, with necrotic depressed centers that were often ulcerated. Based on clinical assessment, cases were classified into mild (23), moderate (51), and severe (21) categories, highlighting the varied presentation and severity of sheeppox among affected sheep.

Blood examination of affected animals demonstrated a notable increase in the mean leukocyte count, reaching 10.88 ± 0.32 × 10^3^/µL, and a reduction in the total erythrocyte count (9.579 ± 0.25 × 10^6^/µL) and hemoglobin levels (7.453 ± 0.22 g/dL) compared to healthy animals (Table 2). The serum biochemical analysis found no significant differences between the control group (healthy animals) and those infected with sheeppox (Table 3).

**Figure 3:**
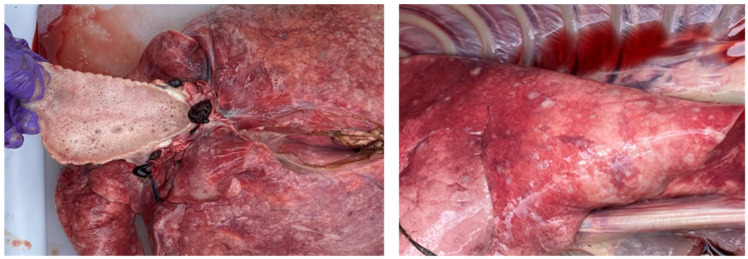
Postmortem examination of confirmed pox-positive cases. The image shows characteristic pox lesions, resembling gunshot wounds, in the nodular stages, distributed throughout the lung parenchyma. Additional findings include edema, congestion, and consolidation.

During the postmortem examination of sheeppox-affected sheep, distinctive pox lesions resembling gunshot wounds were observed in the lungs, along with signs of congestion and hemorrhages (Figure 3). Intestines showed serosal and mucosal congestion with raised small white papules ranging from 0.5 to 3 cm in diameter. The liver was enlarged, and the kidneys revealed discrete, multiple, small white necrotic foci ranging from 0.1 to 0.2 cm. The cut section showed a pale cortex with a congested corticomedullary junction along with a few discrete, small necrotic foci. 

**Figure 4:**
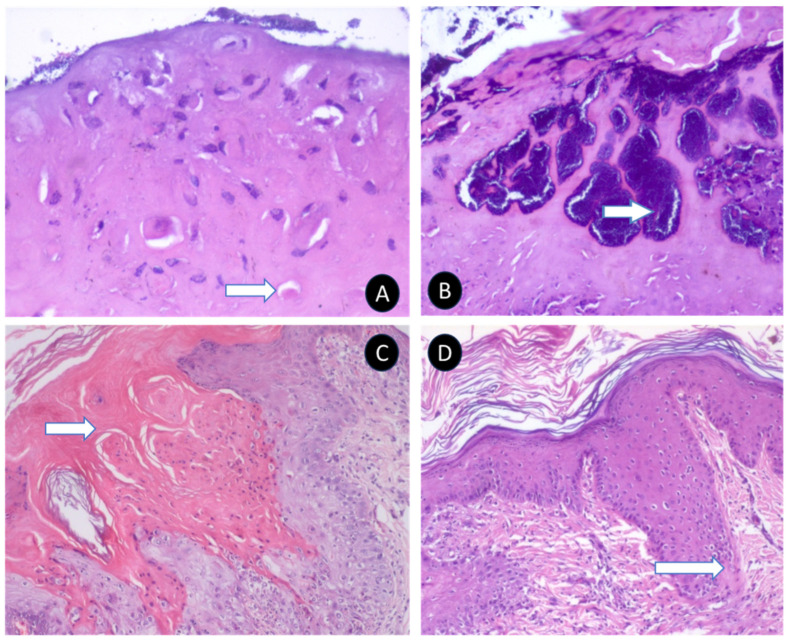
Histopathological changes in various organs due to sheeppox disease. Microscopically, the scabs appear as structureless, homogeneous, coagulated masses (**A**) displaying bacterial colonies and micro-abscesses of varying sizes (**B**). Common observations also include hyperkeratosis (**C**) and epidermal hyperplasia (**D**).

**Figure 5:**
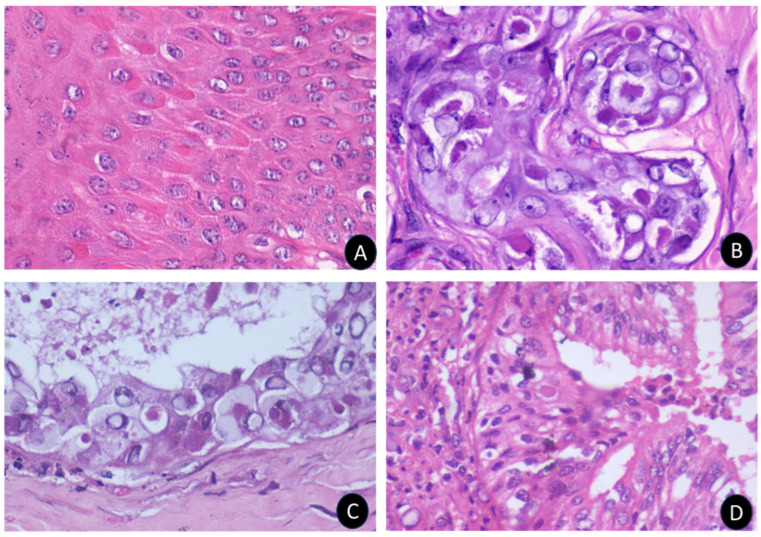
The histopathological changes in different organs of sheeppox disease. The sheeppox cells are identified by a conspicuous intranuclear vacuole with margination of chromatin and inclusion bodies in the skin (**A**), apocrine gland (**B**), epidermal cells (**C**), and lung (**D**) with pneumocytes.

Microscopic examination of scab lesions revealed necrotic cellular debris entrapped within a coagulated mass. Eosinophilic inclusion bodies of various sizes were present in most scabs, along with micro-abscesses, showcasing the localized inflammatory response and viral replication (Figure 4). The macules or papules displayed epidermal acanthosis, hyperkeratosis, and parakeratosis (Figure 4). The hyperplastic epithelium varied in thickness and showed vacuolation in the stratum spinosum layer, with margination of the chromatin and presence of intracytoplasmic eosinophilic inclusions, referred to as sheeppox cells or ‘Cellules claveleuse’ (Figure 5).

In the dermis, perifolliculitis was a notable feature, characterized by the infiltration of either polymorphonuclear or mononuclear cells involving multiple follicles, accompanied by concentric connective tissue proliferation. Many cells in this area contained eosinophilic intracytoplasmic inclusions of various sizes and shapes, further confirming the viral etiology of the lesions (Figure 5).

Nodular lesions showed fibrotic changes and connective tissue proliferation extending into the subcutaneous layer, with hyperemic blood vessels indicating increased blood flow due to inflammation. These lesions also exhibited reparative changes, including complete or incomplete re-epithelialization of the epidermal layer. The fibrotic changes were particularly pronounced around adnexal structures such as degenerating hair follicular bulbs, blood vessels, and apocrine glands.

### 3.3. PCR and Sequencing Analysis of P32 and RPO30 Gene of Sheeppox Virus

In the investigation of 105 cases from 11 suspected sheeppox outbreaks, PCR testing identified 95 samples (90.47%) as positive for Capripoxviruses. These positive samples included scabs (53), nasal swabs (28), ocular swabs (3), and tissues (11), which showed specific amplification of a partial *P32* gene segment approximately 237 bp in size. Further analysis conducted on six representative isolates from the outbreaks in Bengaluru, Chikkaballapura, Kolar, Tumkur, Bellary, and Chitradurga successfully amplified the full length of the *P32* and *RPO30* gene, with an expected amplicon size of approximately 1006 bp and 585 bp, respectively. The complete coding sequences of these SPPV isolates were identified to be 972 and 585 bp in length.

**Figure 6:**
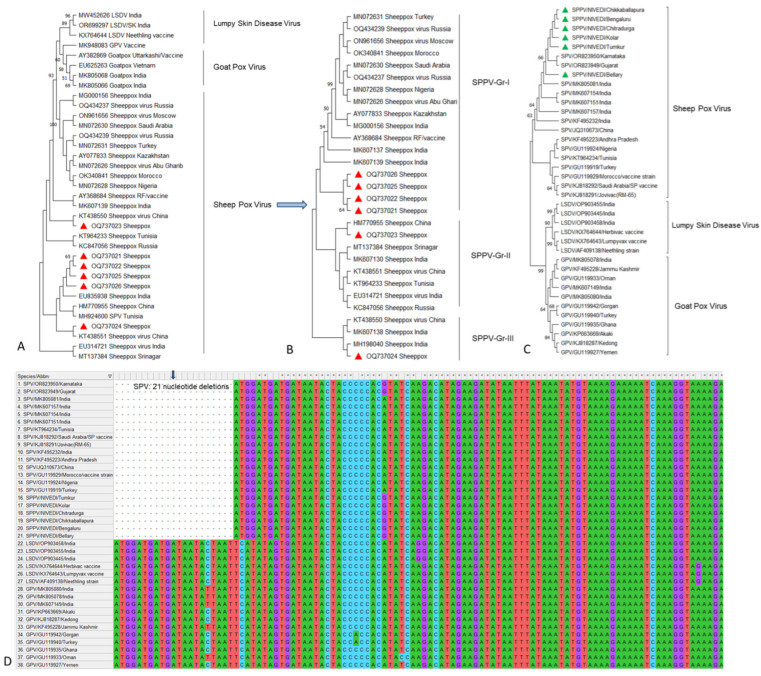
Phylogenetic analysis based on a sequence of full-length *P32* gene of SPPV isolates. The maximum likelihood phylogenetic tree was constructed following MUSCLE alignment in MEGA-X version 10.0.5 [10] with a 1000 bootstrap value for sequence comparison. Under the genus Capripoxvirus, the present isolates formed separate clades as SPPV, compared to GTPV and LSDV based on *P32* gene (**A**,**B**) and *RPO30* gene (**C**) and unique 21 deletion observed in SPPV compared to GTPV (**D**).The present study samples are depicted in red and green color triangles.

The phylogenetic analysis based on the *P32* and *RPO30* genes from SPPV isolates revealed three major lineages within the CaPV isolates: SPPV, GTPV, and lumpy skin disease virus (LSDV) (Figure 6A,C). Among the SPPV, there were three subgroups within and outside India (Figure 6B). Multiple sequence analyses of the Indian *P32* sequences showed a high degree of homology, with 99.5–100% identity at the nucleotide level and 98.1–99.7% identity at the amino acid level among SPPV isolates. Comparisons of SPPV isolates within India demonstrated 98.7–100% nucleotide identity and 98.8–99.7% amino acid identity. The sequence identity of the *RPO30* gene among Indian SPPV isolates showed 98.8–100% and 96.5–99.5% similarity at the nucleotide and amino acid levels, respectively. Whereas, the sequences from other countries showed 92.1–99.8% and 92.6–99.0% similarity at the nucleotide and amino acid levels, respectively. Further, the divergence analysis revealed the highest sequence divergence of 1.2 to 3.2 within the Indian isolates, and 3.7 to 4.8 with other countries at nucleotide and amino acid levels, respectively. Notably, the SPPV isolates from this study shared the highest homology with isolates from neighboring China, indicating a close genetic relationship and potentially similar epidemiological characteristics across these geographic regions. The multiple sequence alignment of the *RPO30* gene from SPPV with GTPV and LSDV isolates revealed the deletion of a unique set of 21 nucleotides (13–33) (corresponding to a seven amino acid sequence), whereas these 21 nts were present in GTPV and LSDV isolates (Figure 6D).

## 4. Discussion

Sheeppox and goatpox, due to their highly contagious nature, impose significant economic burdens on farmers through substantial morbidity and mortality among small ruminants [13]. The persistence of these diseases in endemic regions can be attributed to several factors, including low vaccination coverage, animal migration, environmental conditions, age, sex, breed, nutritional and immunological status of affected animals, and the virulence of circulating viral strains, among others [13].

Our analysis of secondary data of sheep and goatpox outbreaks in Karnataka from 2010 to 2022 highlighted the diseases’ endemic presence across different districts, correlating outbreak occurrence with small ruminant population density. As reported earlier, factors such as seasonal migration, environmental conditions, agent-specific characteristics, and insufficient vaccination efforts have been identified as contributing to these outbreaks [14,15]. The observed high case fatality rates underscore the diseases’ severity and the critical need for preventive vaccination, which, as of now, is not mandated in India’s annual vaccination calendar.

The majority of outbreaks were reported during the colder and warmer months, suggesting that adverse temperatures, malnutrition, water scarcity, and the stresses of migration might compromise animal immunity, leading to disease outbreaks [7,15]. A direct correlation between outbreaks and temperature, and an inverse relationship with rainfall, further supports the impact of environmental factors on disease incidence.

In the present study, we used in-house-developed iELISA to assess whether sheep populations in Karnataka were exposed to sheeppox viruses. The seroprevalence data showed that 3 to 31% of sheep were exposed to SPPV and/or GTPV viruses. This study showed that all the selected study districts had showed seropositivity, which means the disease was widespread in all study zones. However, the rate of seropositivity varied among the districts, as reported from other parts of the world [16,17].

During field visits, it was observed that the shepherds varied in their management practices during the course of disease outbreaks, which might have contributed to the high case fatality rate recorded, along with host, agent, environment, feed scarcity, and inadequate veterinary services, which may directly influence disease outcome [14,15,18]. 

Our study found an overall morbidity rate of 10.69%, with the highest rates in backyard/migratory flocks. This discrepancy can be attributed to more rigorous vaccination programs and prompt treatment protocols in organized farms than in backyard farms. The majority of field outbreaks occurred in unorganized farms, where extensive grazing and herding practices likely facilitated disease spread. The virus’s stability in environmental conditions and its ability to remain viable on contaminated objects and wool for extended periods exacerbate this risk [4,5,14,16,19].

The occurrence of sheeppox has been reported regularly with high morbidity and mortality in sheep irrespective of breed, age, and sex [6,7,15,18,19,20,21,22]. Notably, morbidity and mortality were highest among sheep aged 1–2 years. While lambs are traditionally considered more susceptible [23,24], our findings indicate a significant impact on this slightly older age group, possibly due to a combination of factors, including breed [5,18,22], immunocompromised status, and environmental stresses [25,26,27]. Clinical observations align with previous reports [7,24,28,29,30], highlighting respiratory symptoms, fever, and characteristic skin lesions, which evolve from papules and nodules to vesicles and scabs. The presence of pox lesions in the mucosal surfaces of other internal organs indicates the widespread nature of the infection.

The microscopic examination of sheeppox-affected sheep revealed significant alterations in the skin epithelium, with keratinocytes and subepidermal layers being the primary sites of infection. This led to hyperplasia and granulomatous inflammation, consistent with findings from previous studies [24,28,31,32]. The development of pox lesions across various skin locations can be attributed to the epitheliotropic nature of the virus, which targets epithelial cells, causing pathological changes due to viral replication. The formation of papules, attributed to the proliferation of keratinocytes and subepidermal edema, results in the elevation of these lesions above the level of the surrounding skin. These papules often merge to form firm nodules [7], indicating the presence of both vesicular and nodular forms of sheeppox in the observed cases.

This study suggests the coexistence of the classical vesicular form and the nodular form of sheep pox within Karnataka, aligning with documentation from other regions globally [33]. The observed hyperplastic changes are linked to the virus’s encoding of genes homologous to epidermal growth factor (EGF) proteins [34,35], indicating a potent form of the pox virus epidermal growth factor homolog. This Capripoxvirus’s characteristic epitheliotropic behavior, where it multiplies within the cytoplasm of epithelial cells, underscores the complex interaction between the virus and host cell machinery, leading to the varied clinical manifestations observed in affected animals.

This study recorded instances of sheeppox virus-induced vasculitis, perivasculitis, and perifolliculitis, highlighting the virus’s capacity to induce widespread inflammatory responses in infected sheep [24,31]. Notably, the hair follicular cells displayed significant pathological changes, including severe vacuolization, margination of nuclear chromatin, and the presence of eosinophilic cytoplasmic inclusions, characteristic features of cells infected by the sheep pox virus. These observations are in line with previous reports [36], which similarly identified such cells as indicative of sheeppox infection.

Moreover, this study observed that the sheeppox virus also targets sweat gland epithelial cells, causing vacuolization, the presence of viral inclusions, and cellular necrosis. This extensive impact on various types of skin cells contributes to the diverse clinical manifestations of sheeppox, from localized skin lesions to more systemic signs of infection, and underscores the complexity of the disease’s pathogenesis.

PCR has become a cornerstone in the diagnosis of Capripoxviruses, including those responsible for sheeppox, due to its exceptional sensitivity, specificity, and reproducibility [37]. Among the genes targeted in these diagnostic efforts, the *P32* gene stands out as a structural gene harboring a major viral antigen determinant, which plays a crucial role in the pathogenicity, diagnosis, prevention, and control of Capripoxvirus infections. The utility of PCR in detecting Capripoxviruses is further demonstrated by various studies [6,7,22] that have employed primers to amplify different regions of the *P32* gene, resulting in amplicons ranging from 237 bp to 1010 bp in size.

In this study, phylogenetic analysis and BLAST-n alignment were instrumental in confirming that the strains of the sheeppox virus (SPPV) under investigation all belong to the sheeppox group. Despite the close genomic relationship between SPPV and the goatpox virus (GTPV), with more than 97% similarity, recent molecular research has revealed that the presence of species-specific genes [11,38] and the conservation of unique amino acids within these genes [11,38] enable the differentiation of these viruses into two distinct species. This study has identified the conservation of species-specific residues, highlighting their potential utility in developing molecular markers capable of distinguishing between SPPV and GTPV.

The findings from various studies in India, focusing on mixed flocks often comprising both sheep and goats, have highlighted that outbreaks of sheeppox or goatpox tend to occur only within the respective host species, without cross-species infection [9,14,18]. This specificity is underscored by sequence analysis of the *P32* gene, which revealed that new sheeppox virus (SPPV) isolates exhibited 100% homology with other Indian SPPV isolates across different regions of the country, indicating the circulation of genetically similar virus strains within India.

This study further illuminated that Capripoxviruses (CaPVs) demonstrate a preference for their host species and exhibit lineage specificity, which is based on the origin of the isolates and distinguished by signature residues on the *P32* and *RPO30* genes. This specificity aligns with molecular studies asserting that SPPV and goatpox virus (GTPV) are phylogenetically distinct entities that exhibit clear host preferences [9,11,39,40]. Notably, the majority of characterized CaPV isolates in India have shown this lineage specificity, correlating closely with their host origin, underlining that SPPV infects only sheep and GTPV infects only goats in natural conditions. The *RPO30* gene sequence analyses identified a deletion of 21 nucleotides (seven amino acid sequence) in all Indian and foreign SPPV isolates. Similar to an earlier study [9,11] based on the *RPO30* gene, high sequence similarity among SPPV and with GTPV in India and other countries was recorded, irrespective of geographical region and temporal outbreaks. The genetic findings support either circulation of similar SPPV circulating in India, potentially introduced and evolved from other countries around the world, or else the Indian SPPV isolates may be clonal in nature and distributed to other regions, eventually evolving over a period of time [9,11].

These observations underscore the highly lineage-specific nature of Indian sheeppox viruses, which correlate closely with the host of origin. Unlike other members of the genus Capripoxvirus, the lumpy skin disease virus (LSDV) was reported as jumping the host species [40,41,42,43] in India. In light of these findings, it is recommended to implement a homologous vaccination strategy that utilizes selective host/viral strains to enhance the protection of susceptible animals. Such a targeted approach would be more effective in preventing the spread of sheeppox and goatpox in India.

## 5. Conclusions

The epidemiological study of sheeppox outbreaks has underlined the disease’s high prevalence (seroprevalence 3–31%) and endemic status in the southern region of India. The outbreaks have been associated with a mortality rate of 4.72% in affected flocks and an average case fatality rate of 45.26%, highlighting the significant impact of the disease on small ruminant populations. The phylogenetic analysis, focusing on the full-length *P32* and *RPO30* gene sequencing, has identified the circulation of genetically conserved, species-specific sheeppox virus strains within the region. This finding underscores the necessity of employing homologous vaccines tailored to these specific virus strains for more effective prevention and control measures. However, this study also emphasizes the need for further research, including comparative pathology and additional molecular studies, to enhance our understanding of the increasing species specificity of sheep and goatpox viruses.

## Figures and Tables

**Table 1:** Details of samples collected from different outbreaks in Karnataka.

District	Blocks Affected	Rearing System	Total Number (Susceptible)	Clinically Affected	SPPV-PCR	Morbidity (%)	Mortality (%)	Case Fatality Rate (CFR%)
Chikkaballapura	Gouribidanur	Organized	52	08	08	15.38	03.85	25.00
		Backyard	27	12	10	37.04	14.81	40.00
	Shiddlagatta	Backyard	40	04	03	07.50	07.50	100.0
	Erbachenahalli	Organized	60	08	07	11.67	10.00	85.71
Bengaluru urban	DG Halli	Backyard	17	10	10	58.82	29.41	50.00
Bengaluru rural	Hesaragatta	Backyard	35	03	03	08.57	02.86	33.33
Kolar	Shrinivaspur	Organized	176	07	06	03.41	01.14	33.33
Bellari	Harappanahalli	Organized	160	08	08	05.00	01.88	37.50
	Chigateri	Organized	260	15	14	05.38	01.92	35.71
Tumkur	Sira	Backyard	16	13	11	68.75	37.50	54.55
Chitradurga	Hosadurga	Backyard	46	17	15	32.61	10.87	33.33
Total			889	105	95	10.69	04.72	45.26

**Table 2:** Mean (±SE) values of hematological parameters in the control and affected animals with sheep pox.

Hematology Parameters	Control Sheep (n = 6)	Sheeppox Positive (n = 71)
TLC (×10^3^ /µL)	6.335 ± 0.2871	10.88 ± 0.3191 ***
TEC (×10_6_ /µL)	11.49 ± 0.5396	9.579 ± 0.2493 *
Hb (g/dL)	9.355 ± 0.3703	7.453 ± 0.2242 *
HCT (%)	30.98 ± 0.5605	23.36 ± 0.6026 ***
PLT (105 /µL)	2.475 ± 0.1817	2.964 ± 0.2142

Hematology parameters: control (n = 6), sheeppox positive (n = 71). *—Significant at *p*< 0.05 ***—highly significant at *p* < 0.001.

**Table 3:** Mean ± SE values of biochemical parameters in control and affected animals with sheeppox.

Biochemical Parameters	Control Sheep (n = 6)	Sheeppox Positive (n = 71)
AST (U/I)	157.8 ± 15.29	234.8 ± 18.48
ALP (U/I)	175.5 ± 15.53	296.0 ± 26.97
Total Protein (g/dL)	6.578 ± 0.2140	6.967 ± 0.3505
Albumin g/dL	3.967 ± 0.1687	4.293 ± 0.2336
GGT (U/I)	57.27 ± 3.560	65.06 ± 2.057
ALT (U/I)	29.64 ± 1.053	29.68 ± 3.380
Creatinine (mg/dL)	1.005 ± 0.1132	1.002 ± 0.09801

## Data Availability

The data presented in this study are available on request from the corresponding author. The data are not publicly available due to the laid down rules of the institute.

## Supplementary Materials

The following supporting information can be downloaded at https://www.mdpi.com/article/10.3390/microorganisms12071373/s1, Figure S1: Cumulative sheepox disease outbreaks: the bar diagram showing the cumulative number of sheeppox disease outbreaks in different districts of Karnataka state in India (2010–2022); Figure S2: temporal distribution of sheepox: the bar diagram showing the cumulative number of sheeppox disease outbreaks and deaths over a period of 12 years in Karnataka state of India.
