# Supplementary material for: Epidemiological, Pathological, and Molecular Studies on Sheeppox Disease Outbreaks in Karnataka, India
Source: Microorganisms. 2024 Jul 4;12(7):1373. doi: 10.3390/microorganisms12071373 (PMC11279338; doi:10.3390/microorganisms12071373)
Supplement: Supplementary file 1 [file microorganisms-12-01373-s001.zip › microorganisms-2908340-supplementary.pdf]

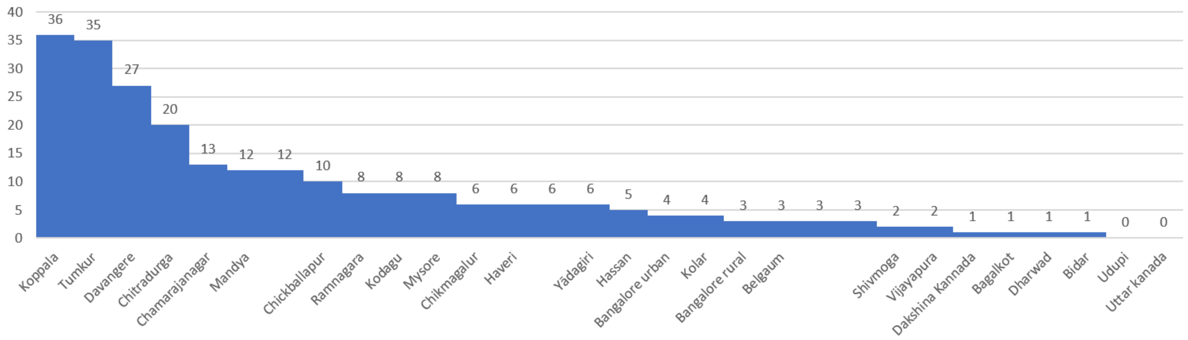

Figure S1: Cumulative sheepox disease outbreaks: The bar diagram showing the cumulative number of sheeppox disease outbreaks in different districts of Karnataka state in India (2010-2022).

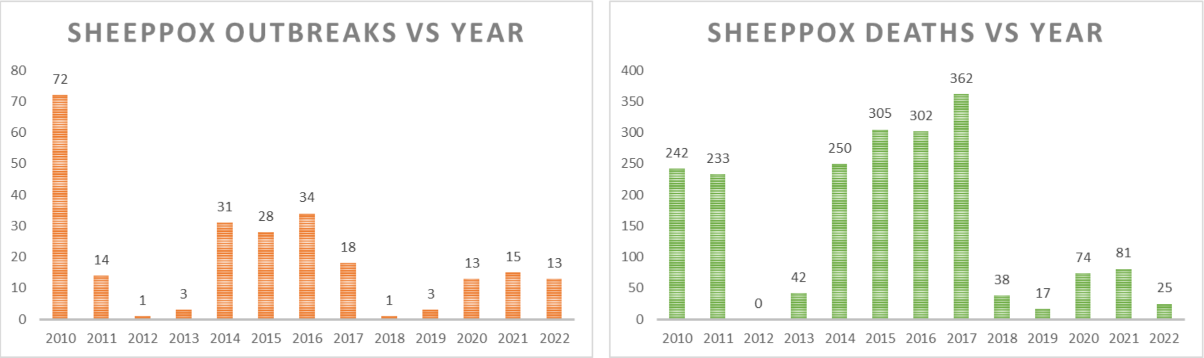

Figure S2: Temporal distribution of sheepox: The bar diagram showing the cumulative number of sheeppox disease outbreaks and deaths over a period of 12 years in Karnataka state of India.
